# Impact of contouring variability on oncological PET radiomics features in the lung

**DOI:** 10.1038/s41598-019-57171-7

**Published:** 2020-01-15

**Authors:** F. Yang, G. Simpson, L. Young, J. Ford, N. Dogan, L. Wang

**Affiliations:** 1 Department of Radiation Oncology, University of Miami, Miami, FL USA; 2 Department of Biomedical Engineering, University of Miami, Miami, FL USA; 3 Department of Radiation Oncology, University of Washington, Seattle, WA USA

**Keywords:** Cancer imaging, Tumour biomarkers

## Abstract

Radiomics features extracted from oncological PET images are currently under intense scrutiny within the context of risk stratification for a variety of cancers. However, the lack of robustness assessment poses problems for their application across institutions and for broader patient populations. The objective of the current study was to examine the extent to which radiomics parameters from oncological PET vary in response to manual contouring variability in lung cancer. Imaging data employed in the study consisted of 26 PET scans with lesions in the lung being created through the use of an anthropomorphic phantom in conjunction with Monte Carlo simulations. From each of the simulated lesions, 25 radiomics features related to the gray-level co-occurrence matrices (GLCOM), gray-level size zone matrices (GLSZM), and gray-level neighborhood difference matrices (GLNDM) were extracted from ground truth contour and from manual contours provided by 10 raters in regard to four intensity discretization schemes with number of gray levels of 32, 64, 128, and 256, respectively. The impact of interrater variability in tumor delineation upon the agreement between raters on radiomics features was examined via interclass correlation and leave-*p*-out assessment. Only weak and moderate correlations were found between segmentation accuracy as measured by the Dice coefficient and percent feature error from ground truth for the vast majority of the features being examined. GLNDM-based texture parameters emerged as the top performing category of radiomcs features in terms of robustness against contouring variability for discretization schemes engaging number of gray levels of 32, 64, and 128 while GLCOM-based parameters stood out for discretization scheme engaging 256 gray levels. How and to what extent interrater reliability of radiomics features vary in response to the number of raters were largely feature-dependent. It was concluded that impact of contouring variability on PET-based radiomics features is present to varying degrees and could be experienced as a barrier to convey PET-based radiomics research to clinical relevance.

## Introduction

Lung cancer has the highest mortality rate of all cancers for both men and women with a predicted 5-year survival rate of 8–13%^1^. To maximize positive outcomes, cancer treatment is evolving towards personalized care by evaluating predictive factors before treatment and disease response while treatment is occurring^2,3^. ^18^F-fluoro-deoxy-2-glucose (^18^F-FDG) positron emission tomography (PET) is increasingly becoming the standard of care in baseline staging and restaging of lung cancer as well as treatment monitoring and tracking response to treatment in radiation therapy (RT)^4,5^. As a functional imaging modality, FDG-PET offers the unique ability to provide the metabolic characterization of tumoral microenvironment not afforded by other imaging modalities such as computed tomography (CT) and conventional magnetic resonant imaging (MRI). Radiomics analysis of FDG-PET imaging data in RT seeks to quantify intratumoral heterogeneity under the assumption that radiotracer accumulation pattern within the tumor is associated with the spatial phenotypic variation in metabolic traits of the cancer and thus potentially furnishes predictive information concerning disease recurrence and responsiveness to therapeutics.

Previous studies on PET radiomics for lung cancer have linked PET radiomics parameters to various clinical end points including overall survival, disease specific survival, and locoregional control amongst others^6–10^. Successful translation of PET radiomics research into the clinic for lung cancer management in RT hinges largely on the robustness of radiomics parameters to various inter/intra-scanner image acquisition variability as well as against an array of diverse uncertainties intrinsic to radiomics analysis. Concerns of reproducibility and reliability have been investigated by several studies with emphasis being put, respectively, on the impact onto PET radiomics features due to factors including PET scanner manufactures, acquisition and reconstruction algorithms, intensity discretization scheme, *etc*.^11–14^. As for the variability of contouring volume of interest (VOI) for inclusion to radiomics analysis, only a few existing studies investigated its impact on PET radiomics parameters^15,16^, even though contouring variability is commonly recognized as the largest source of error in the RT planning process^17^. These studies involving contouring were carried out using clinical PET imaging data with an inherent lack of knowledge about actual tumor volumes. Without this knowledge, variance of PET radiomics parameters with respect to contouring variability can be assessed to only a very limited degree while the extent to which radiomics parameters deviate from their true values due to contouring variability remains largely unaddressed. By aid of a realistic 3D digital phantom of the thorax in conjunction with a Monte Carlo (MC) based PET imaging simulation package, the purpose of this work was to examine the impact of contouring variability on PET radiomics parameters for lung cancer in a setting with complete knowledge of tumor location, morphology, and intensity distribution. By so doing, the present work furnished a set of methodological guides exploitable for design and implementation of future investigations endeavoring to translate PET radiomics into clinical relevance.

## Materials and Methods

### PET image simulation

A digital phantom was utilized for the generation of PET data. The rationale behind using a digital phantom was that the digital phantom provides image data with a known association to the ground truth and therefore offers an objective criterion for inclusion and exclusion of image content from radiomics analysis. For the simulation, the Zubal anthropomorphic phantom^18^ was used as the attenuation map and the Monte Carlo based Simulation System for Tomography software package (SimSET)^19^ was employed for PET event detection process. The PET system modeled was a Siemens Biograph scanner featuring a pixelated block BGO detector with a ring radius of 42.1 cm. The emission data produced from the simulations was re-binned into 128 × 128 sinograms by single-slice re-binning, followed with reconstruction using an ordered subset expectation maximization (OSEM) algorithm (8 iterations, 4 subsets). Attenuation correction was conducted using tissue-specific indices that correspond to attenuation coefficients defined in SimSET. The resulting image data was further convolved with a 5 mm full width at half maximum (FWHM) 3D Gaussian filter for noise suppression. The methodology along with the full details used for generating PET imaging data was previously published and validated by the demonstrated close agreement from comparison of the actually acquired and the simulated image data in terms of gray-level intensity histogram, intensity profile, and statistical textures^20–23^.

### Volume contouring and evaluation

A total of 10 radiation oncology physicians (*i*.*e*., raters) with extensive clinical experience on PET-based lung lesion delineation participated in the study. The software platform used for contouring was MIM Maestro v6.5.5 (MIM, Software, Cleveland, OH). Raters were given no specific directions regarding the display settings such as window/level, thresholding, and pixel representation amongst others. Allowed for use were only the fundamental contouring tools of the MIM Maestro program including brush, pen, interpolation, and smoothing, *etc*. Accuracy of manual contours relative to their respective ground truth was assessed through use of Dice coefficient (*DICE*)^24^ and symmetric mean absolute surface distance (*SMASD*)^25^. *DICE* can have a range between 0 and 1, with 0 indicating a manual contour does not overlap spatially with its ground truth while 1 indicating that the two match identically. *SMASD* estimates the spatial distance between a manual contour and its ground truth through quantifying the average extent in terms of voxel size to which the surfaces of the two differ. Ethical approval was not applicable for the current study according to US Health and Human Services regulations (45 CFR part 46)^26^, because the contour data contributed by the raters was anonymized and no rater information might be individually identifiable.

### Radiomics analysis

For each of the simulated lesions, radiomics analysis was performed using each rater’s VOI as well as the ground truth volume. Given the fact that it has not been established what discretization method is most suited for PET-based radiomics feature extractions^27^, the current study took into consideration four of the most frequently used discretization schemes with image intensity values inside the VOIs for radiomics analysis being rescaled to the range of integers [0, 31], [0, 63], [0, 127], and [0, 255], respectively. The radiomics parameters evaluated included an array of the most commonly referenced volumetric texture features related to the gray-level co-occurrence matrices (GLCOM) with voxel displacement of 1, gray-level size zone matrices (GLSZM), and gray-level neighborhood difference matrices (GLNDM) with neighborhood size of 3 × 3 × 3^28–30^. GLCOM-based features quantify the frequency of a voxel intensity pattern relative to another at a given distance. For example, GLCOM-based feature contrast quantifies the frequency of co-occurring gray level intensities by summing over all gray level combinations and weighting the probability of each occurrence by their difference, *i*.*e*. larger differences between two voxels are weighted more heavily than similar gray level intensities. A higher value of contrast is indicative of a GLCOM which is associated with low probabilities of similarly occurring gray level intensities. GLSZM-based features either accentuate the size of isointense gray level regions or emphasize high or low gray level intensity regions. For example, small zone emphasis weights the smaller regions of isointense gray levels higher than larger zones. GLNDM-based features describe how the gray level intensities around a certain gray level differ. For example, the GLNDM-based feature contrast describes local neighborhood intensity differences, *i*.*e*., a VOI in which each voxel’s neighborhood has large grayscale intensity difference would have a higher GLNDM-based contrast value. In total, 25 radiomics features were included in the study and for a complete list of these features please refer to Table 1.

**Table 1:** Radiomics feature classes and features being examined for dependency on contouring variability.

Category	Feature
Gray-level Co-occurrence Matrix (GLCOM)	Energy
	Contrast
	Entropy
	Homogeneity
	Correlation
	Variance
	Dissimilarity
Gray-level Size Zone Matrix (GLSZM)	Short Zones Emphasis (SZE)
	Large Zones Emphasis (LZE)
	Gray-level Non-uniformity (GLN)
	Zone Size Non-Uniformity (ZSNU)
	Zone Percentage (ZP)
	Low Gray-level Zones Emphasis (LGZE)
	High Gray-level Zones Emphasis (HGZE)
	Short Zones Low Gray-level Emphasis (SZLGE)
	Short Zones High Gray-level Emphasis (SZHGE)
	Large Zones Low Gray-level Emphasis (LZLGE)
	Large Zones High Gray-level Emphasis (LZHGE)
	Gray-level Variance Emphasis (GLV)
	Zone Size Variance Emphasis (ZSV)
Gray-level Neighborhood Difference Matrix (GLNDM)	Coarseness
	Contrast
	Busyness
	Complexity
	Strength

### Statistical analysis

Statistical analysis was performed using JMP Pro® Version 12 (SAS Institute Inc., Cary, NC) statistical software. Percent error between feature value derived from raters’ VOI and its ground truth value was calculated and examined for correlation with *DICE* to investigate the dependence of feature uncertainty on contouring accuracy through use of Spearman’s rank correlation coefficient ($\rho$). Correlations were considered weak if $|\rho |$ < 0.400, moderate if 0.400 ≤  $|\rho |$ < 0.600, relatively strong if 0.600 ≤  $|\rho |$ < 0.800, and strong if 0.800 ≤  $|\rho |$^31^. To measure the impact of contouring variability on PET radiomics parameters, the intraclass correlation coefficient ($ICC$) was calculated using the two-way random effects model^32^. This model estimates the absolute agreement of multiple raters per measurement. Higher agreement indicates a lower dependence of a given feature upon contouring variability. This analysis was performed for each of the 25 radiomics features being investigated. Selection of radiomics features least impacted by contouring variability was based on the lower confidence interval of $ICC$ ($IC{C}_{LB}$) for all 10 raters and a threshold of ≥0.950 (*i*.*e*., an error of less than 5% for worst case scenario) was used as the cutoff value^33^. The impact of decreasing the number of raters for the top performing features was explored by employing a leave-*p*-out analysis^34^. This method calculated the mean lower confidence bound of the $ICC$ coefficient (${\overline{ICC}}_{LB}$) by excluding $p$ number of raters ($1\le p\le 7$) and averaging the lower 95% confidence bound of all remaining rater combinations. For all statistical analyses, $p$-values of 0.050 or lower were considered statistically significant.

## Results

**Figure 1:**
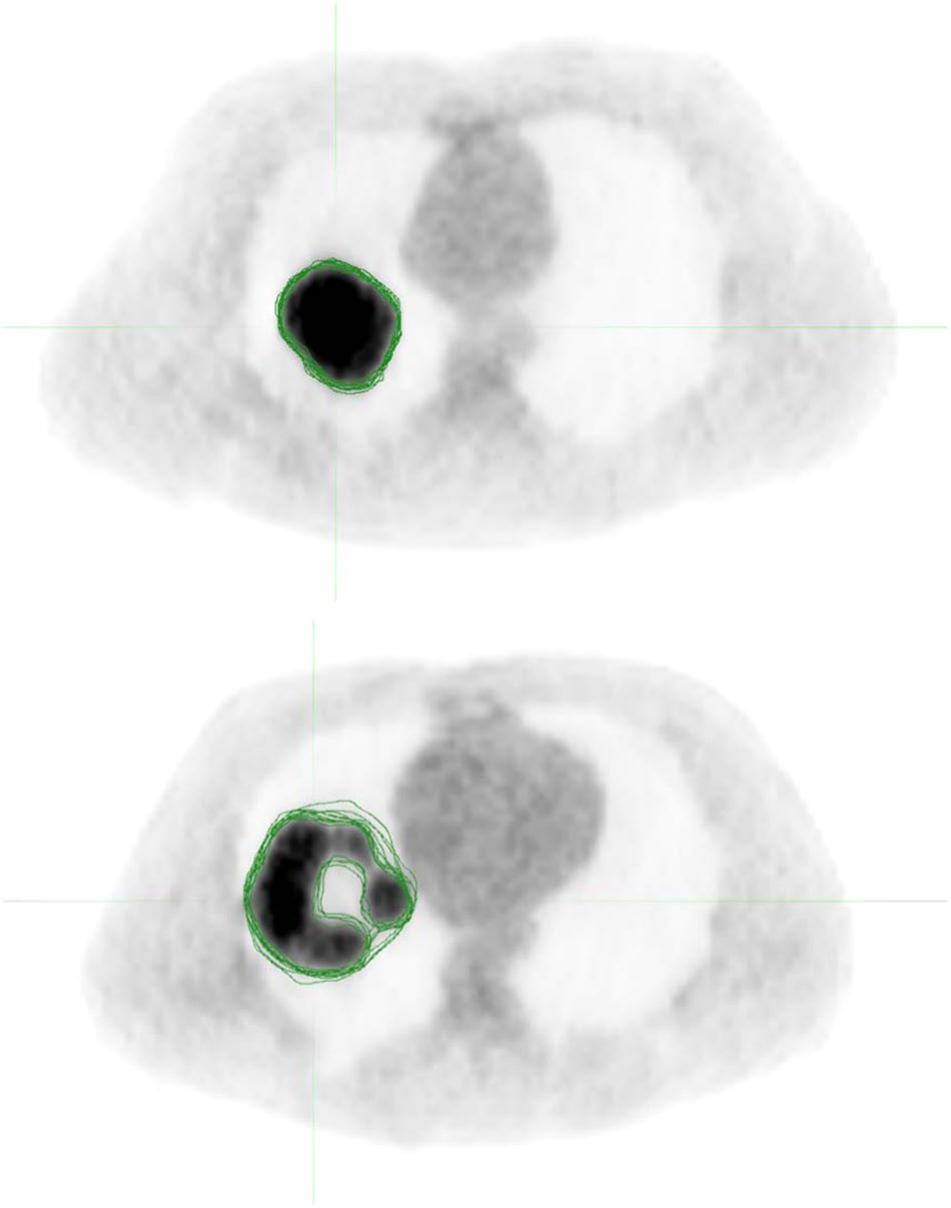
Axial cross-sections of two simulated PET images with the manual contours by the raters overlaid. Rater agreement for the lesion on the top is higher than that for the more complex lesion on the bottom.

**Figure 2:**
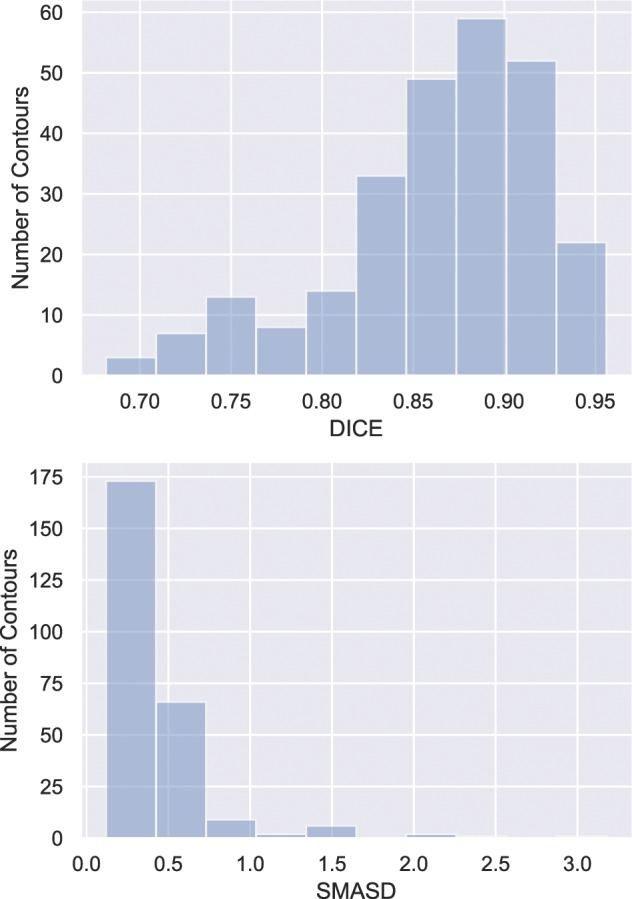
Histogram of $DICE$ (top panel) and $SMASD$ (bottom panel) for all 260 manually defined contours (26 lesions contoured by 10 raters).

A total of 26 lesions were simulated with wide variations in size, shape, and radiotracer uptake pattern as well as anatomical location, ranging from within the lungs to adjacent to the mediastinum or to the chest wall. The ground truth volumes of the simulated lesions ranged from 29.8 cm^3^ to 345.1 cm^3^ while the volumes defined by the raters ranged from 33.4 cm^3^ to 425.7 cm^3^. The volume error averaged over all raters ranged from 19.3 cm^3^ smaller than the ground truth volume to 17.2 cm^3^ larger, with an average error over all tumors of 2.4 cm^3^ larger. Figure 1 illustrates the variability of the raters in delineations of two of the simulated lesions, and the overall variations across the raters are reflected in the distribution of $DICE$ and $SMASD$ values as shown in Fig. 2, from which can be seen that most contoured volumes achieved $DICE$ above 0.800 while $SMASD$ less than 0.550 voxel. It demonstrates that, overall, manual contours by the raters attained substantial spatial overlap with their respective ground truth volumes while approximating their respective ground truth surface within subvoxel accuracy. In addition, contouring performance of the raters was lesion-dependent and did not appear to be associated with any clear and consistent trends allowing to distinguish the raters.

**Figure 3:**
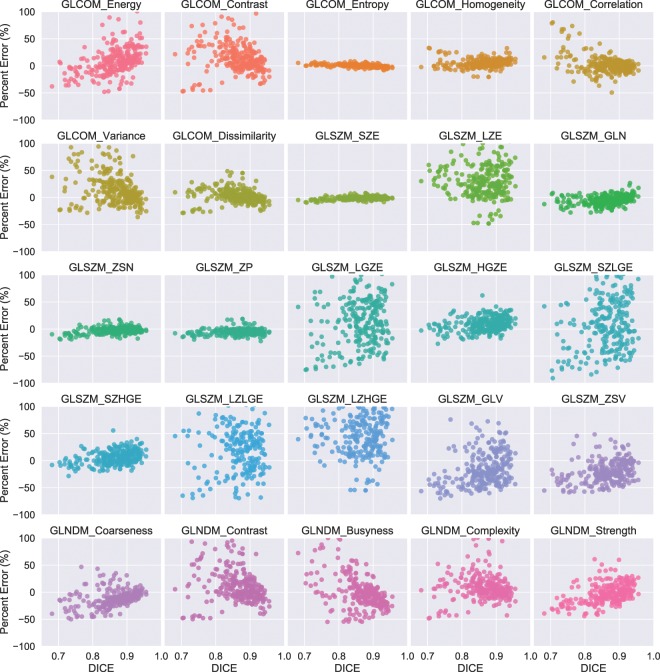
Scatter plots comparing percent error from ground truth against $DICE$ for radiomics features extracted using discretization scheme with number of gray levels of 64.

**Figure 4:**
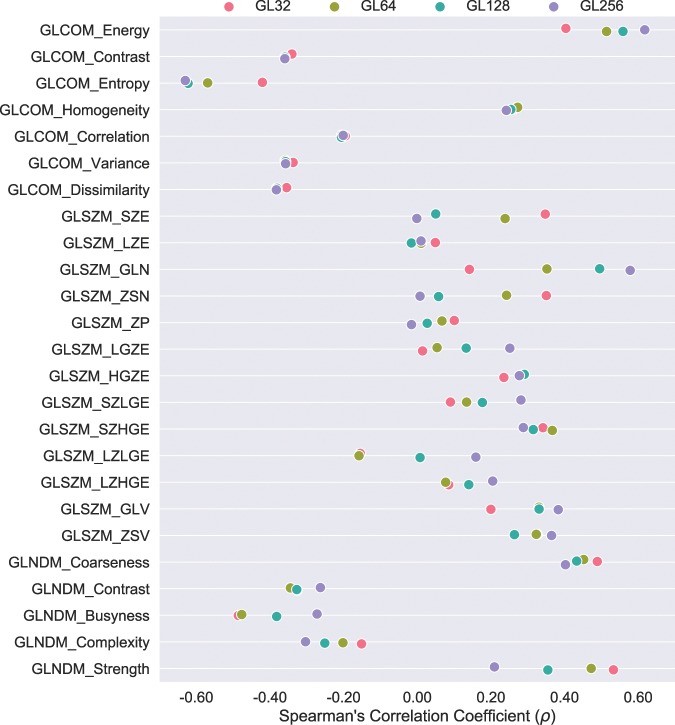
Spearman’ correlation coefficient ($\rho$) between $DICE$ and percent error from ground truth for the studied radiomics features. Results are color coded pink, green, cyan, and purple representing feature extraction done using discretization scheme with gray levels of 32 (GL32), 64 (GL64), 128 (GL128), and 256 (GL256), respectively.

Fig. 3 shows the scatter plots between the percent error of the examined radiomics features from their corresponding ground truth and $DICE$ for discretization scheme with gray levels of 64. As can be readily observed, the patterns between percent feature error and $DICE$ are largely feature-dependent and could vary widely, from percent feature error being relatively robust against contouring accuracy such as for features including GLCOM_Entroy, GLSZM_SZE, GLSZM_ZSN, and GLSZM_ZP, to being very spread out such as for features including GLSZM_LGZE, GLSZM_SZLGE, GLSZM_LZLGE, *etc*., with the rest seen as being mild. Further, it is worth noting that similar tendencies were also observed for the rest of discretization schemes that resulted in intensity values being rescaled to have number of gray levels of 32, 128, and 256. The results of the Spearman’s $\rho$ calculation of correlation between percent feature error and $DICE$ revealed only weak and moderate correlations irrespective of the discretizaiton methods for almost all of the features being examined, except for GLCOM_Entropy extracted with discretizaiton levels of 128 and 256 and GLCOM_Energy with discretizaiton levels of 256. Percent feature error of these three and $DICE$ were correlated relatively strongly with $\rho$ of −0.6277, −0.6303, and 0.6177, respectively. All calculated correlations between percent feature error and $DICE$ for the studied discretization algorithms are presented in Fig. 4.

**Figure 5:**
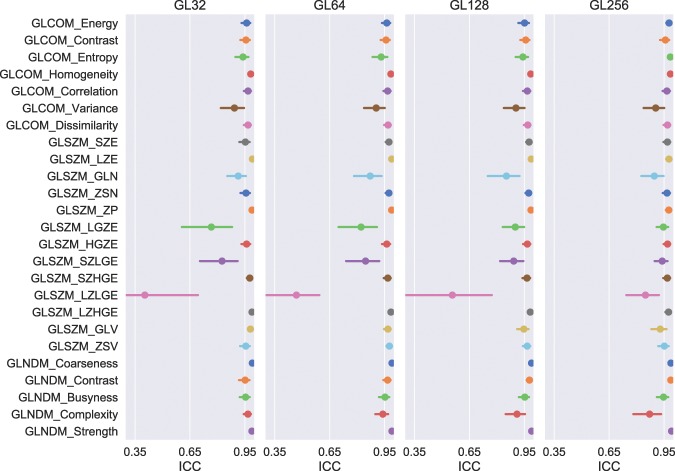
*ICC* with 95% confidence intervals for the studied radiomics features. Panels from left to right for results with feature extraction done using discretization scheme with number of gray levels of 32 (GL32), 64 (GL64), 128 (GL128), and 256 (GL256), respectively. Features with lower bound of confidence interval no less than 0.950 were considered for inclusion in leave-*p*-out analysis.

The results of the $ICC$ analysis performed for each of the features studied are shown in Fig. 5. For the four intensity discretization schemes engaging number of gray levels of 32, 64, 128, and 256, $ICC$ of GLCOM-based features ranged from 0.893 to 0.982, 0.904 to 0.984, 0.904 to 0.985, and 0.904 to 0.985 with mean of 0.952, 0.954, 0.955, and 0.963, respectively. $ICC$ of the 13 GLSZM-based features attained an average of 0.897, 0.887, 0.919, and 0.947 while with range extending from 0.404 to 0.991, 0.468 to 0.989, 0.557 to 0.988, and 0.848 to 0.977 for the four discretization schemes, respectively. As to the five GLNDM-based features, $ICC$ spanned from 0.951 to 0.991, 0.940 to 0.990, 0.910 to 0.990, and 0.871 to 0.991 while averaging 0.970, 0.968, 0.964, and 0.957 for the four discretization schemes, respectively. What was being observed furthermore is the dependence of $ICC$ on the number of the discretization levels. This association was confirmed to be strong for 17 of the 25 features being examined when using Spearman’s rank correlation test. $ICC$ ascended with the number of the discretization levels for nine of the 17 features including GLCOM-based Entropy, Homogeneity, and Variance, GLSZM-based LGZE, HGZE, SZLGE, and LZLGE, and GLNDM-based Contrast and Strength while descending for the other eight consisting of GLCOM-based Correlation, GLSZM-based LZE, ZP, LZHGE, and GLV, and GLNDM-based Coarseness, Busyness, and Complexity.

**Figure 6:**
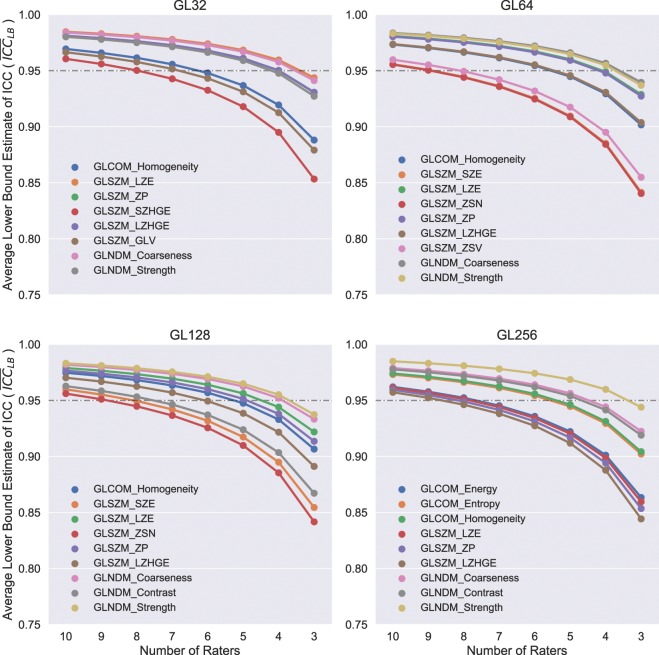
Results of leave-*p*-out analysis for radiomics features with lower bound of confidence interval of $ICC$ computed based on all 10 raters no less than 0.950. Panels from top left to bottom right for results with feature extraction done using discretization scheme with number of gray levels of 32 (GL32), 64 (GL64), 128 (GL128), and 256 (GL256), respectively.

Selection of the top performing features for leave-*p*-out analysis was based upon $IC{C}_{LB}$ and features with $IC{C}_{LB}$ for all raters no less than 0.950 were selected. The number of features qualified by this criterion was eight for the discretization scheme involving 32 gray levels while nine for the rest of the discretization schemes. Worth noting are features consisting of GLCOM-based homogeneity, GLZSM-based LZE, ZP, and LZHGE, and GLNDM-based Coarseness and Strength as they met the criterion regardless of type of discretization method. Results of the leave-*p*-out analysis for these selected radiomics features are showed in Fig. 6, from which it can readily appreciated that as the number of raters was decreased ${\overline{ICC}}_{LB}$ declined, as might be expected, whereas how and the extent to which it declined in response to the number of raters varied for each of the selected features and also depended on the discretization schemes. Among the selected features for the discretization scheme with 32 gray levels, GLSZM-based LZE and LZHGE along with GLNDM-based Coarseness appeared to be the top three most stable ones with the number of raters decreasing. The top three most robust features for the discretization schemes with 64 and 128 gray levels were the same and consisted of GLNDM-based Coarseness and Strength together with GLSZM-based LZE. As to the discretization scheme with 256 gray levels, GLNDM-based Strength performed the best against the number of raters decreasing following by GLNDM-based Coarseness and Contrast.

## Discussion

As the tumor shape increases in complexity and irregularity, the interpretation of lesion border requires raters to make increasingly subjective choices, leading to delineation differences and errors. A general observation based on the calculated $DICE$ and $SMASD$ values was that lesions with simple geometry tended to have higher agreement among manual contours whereas lesions with complex and irregular shapes produced lower agreement (*e*.*g*., see Fig. 1). Use of the ground truth volume information of the lesions allowed quantification of the accuracy and variability of manual contouring and furthermore their impacts on radiomics parameters. The results of only weak and moderate correlations existing between percent feature error and $DICE$ (Figs. 3 and 4) for the majority of the radiomics features being studied implies a complex relationship between volume delineation and feature values. Radiomics features are complex and are related to the gray-level intensities included as well as their arrangement. Two different delineations may attain the same $DICE$ in regard to the ground truth volume while potentially containing different gray-level intensities and arrangements. The observed widely varying patterns between percent feature error and $DICE$ are thus most likely attributed to the reliance of radiomics features on the content of a VOI whereas $DICE$ more on its spatial placement.

The $$ICC$$ analysis afforded an estimate of the reliability of radiomics feature values in the context of delineation variability. Using the McGraw convention, $$ICC < $$ 0.750 is considered poor reliability, within the range of 0.750 ≤ *ICC* < 0.900 good reliability, and 0.900 ≤ *ICC* is considered to have excellent reliability^32^. Upon initial calculation of the $$ICC$$, a majority of the 25 investigated features (21 for the discretization schemes with 32 and 64 gray levels; 22 for the discretization schemes with 128 and 256 gray levels) had $$ICC$$ no less than 0.900. When the number of raters dropped to three, a typical upper limit for number of raters for VOI contouring as seen in majority of radiomics studies, the number of features attaining excellent reliability regressed to no greater than six for any of the discretization schemes, implying the necessity of taking into consideration contouring variability for the design and implementation of PET radiomics studies in future.

Our analysis fits well with previous works and expands on many points. Johnson *et al*. correlated intensity, geometric, and GLNDM-based texture features extracted from synthetic PET images to six contouring accuracy metrics^23^. In agreement with our results, they found a weak correlation between $DICE$ and any of the GLNDM-based features using Pearson’s correlation coefficient. In the present work, correlation between contouring accuracy and texture features was expanded by examining the error from ground truth for three classes of texture features. $ICC$ has been previously used to demonstrate intrarater reliability and reproducibility of texture features, conveniently allowing direct comparison. Leijenaar *et al*. investigated the stability of radiomics features by analyzing test-retest and inter-observer variability of manually delineated Non-Small Cell Lung Cancer (NSCLC) tumors on fused PET-CT data sets^15^. In their study, five physicians contoured 27 lesions from which GLCOM-based features were calculated. It was reported that GLCOM-based Contrast, Homogeneity, and Dissimilarity performed well in regard to stability in inter-observer testing, achieving similar values of *ICC* ≥ 0.900. Bashir *et al*. investigated the effects of segmentation algorithms on PET-derived texture features including manually defined NSCLC lesions^16^. Their study employed three physicians to contour 53 patients. Overlapping metrics included GLCOM-based Homogeneity and Dissimilarity with $ICC$ of 0.782 and 0.753 respectively. Based upon three raters, in our study, the same features displayed low dependence upon contouring variability with average $ICC$ of 0.848 for Homogeneity and 0.850 for Dissimilarity. Although Bashir *et al*. investigated GLNDM-based contrast as well, they applied a logarithmic transform to the feature, rendering direct comparison inappropriate. In addition, previous work investigating the influence of delineation variability on stability of CT-based radiomic features found a strong dependence on cancer site, with relatively high stability for lung cancer^35^. Prior to our study we had no expectation that PET-based radiomic stability results would be similar to results of studies from other imaging modalities. In fact, the two studies result in very similar stability results, with both showing a number of radiomics texture features stable (*ICC* > 0.800) for lung cancer; however, while the CT study showed very high correlation between $DICE$ and stability, the current study showed it is largely feature-dependent. Thus, care should be taken when applying such results across imaging modalities.

The results from the current study present several implications regarding the use of PET radiomics analysis in lung cancer that are worthy of mentioning. Firstly, the connotation that the impact of contouring variability on radiomics parameters is feature-dependent and can vary substantially calls for prudence in the use of PET radiomics parameters in the context of disease classification or risk stratification for lung cancer. A large number of the previous studies in this line of research were carried out with using contouring data from a single or only very few raters and thus conclusions drawn from these studies may potentially be biased by the availability and source of the contouring data. As to future studies on this topic, it is advised that study design should take into account the demonstrated impact of contouring variability upon radiomics parameters so that the effect can be diminished, if not removed entirely. Additionally, the findings also advocate the notion that predictive models based on PET radiomics aiming at supporting the decision-making process for patient risk assessment and treatment response identification, in lung cancer at least, should be introduced with discretion given the well anticipated contouring variability between the training data and the clinical data to be stratified. Despite these caveats for now, however, the outlook is promising for PET radiomics with the likely advent of valid automatic approaches for the segmentation of PET imaged target volumes in near future. By which time, PET radiomics — especially when plugged into the framework of big data analytics — may fulfil the potential of delivering greatly improved diagnostic, prognostic, and predictive accuracy for the personalized management of cancer via harvesting ever increasingly voluminous and heterogeneous imaging data while coming along with the ease of implementation and deployment for clinical use.

Although the current work is informative to the development and translation of PET radiomics research into potential solutions clinically applicable across distinct institutions and for broader populations and is to our knowledge the first attempt to determine how contouring variability implicates radiomics analysis in a setting with complete knowledge of ground truth, there are several limitations that need to be acknowledged. Chief among them is that the raters participating in the study were from a single institution. As such, we were only able to assess the extent of variability in manual contouring PET positive lung targets together with its effects on radiomics parameters in our own setting and the results may not be representative of broader clinical practices in RT. Secondly, this work comprised a rather limited sample size, *i*.*e*., the number of PET lesions being used, which likely had precluded our ability to identify potential associations that might be present as well compromised generalizability of the demonstrated findings. Thirdly, among the wide array of uncertainties and variabilities involved in the processes of image acquisition, pre-processing, and feature extraction for PET radiomics analysis, the current work put emphasis primarily on the influence on radiomics parameters due to contouring variability. Thus, its synergistic effect with those other factors in PET radiomics analysis remains to be further investigated. Furthermore, the current study focused solely on the assessment of the impact on radiomics analysis from variability of manual contouring, owing largely to the fact that, in essence, a valid and convincing automatic “solution” has not been reached so far for the segmentation of PET-imaged tumor volumes^36,37^. As to how and to what extent automatic contouring methods affects oncological PET radiomics analysis, it remains to be dealt with upon the arrival of paradigm shift of PET target volume segmentation from manual to automatic in clinical practices.

## Conclusions

It is apparent from the results of the current study that the impact of contouring variability on PET-based radiomics features varies widely and could be experienced as a barrier to convey PET radiomics research across different institutions and for broader patient populations. It calls for caution in the use of predictive models involving PET radiomics features implicated with contouring variability within the context of disease stratification and risk assessment for lung cancer.

## Data Availability

The datasets used and/or analyzed in the current study are available from the corresponding author upon reasonable request.
